# Mental health and suicide literacy among school nurses in Japan: A cross-sectional study

**DOI:** 10.1371/journal.pone.0334488

**Published:** 2026-06-05

**Authors:** Ayuko Yukawa, Sakurako Kusaka, Satoshi Yamaguchi, Takuya Arai, Fumika Sawamura, Fumiharu Togo, Tsukasa Sasaki

**Affiliations:** 1 Department of Physical and Health Education, Graduate School of Education, The University of Tokyo, Tokyo, Japan; 2 Research Fellow of Japan Society for the Promotion of Science, Tokyo, Japan; 3 Unit for Mental Health Promotion, Research Center for Social Science & Medicine, Tokyo Metropolitan Institute of Medical Science, Tokyo, Japan; 4 Saitama Prefectural Education Bureau, Student’s Consultant Division, Saitama, Japan; 5 Saitama Prefectural Education Bureau Health and Physical Education Division, Saitama, Japan; The Hong Kong Polytechnic University, HONG KONG

## Abstract

**Background:**

School nurses (SN) are key providers of school health services and play a vital role in promoting adolescent mental health and preventing suicide. However, research into their mental health literacy (MHL) and suicide literacy remains limited.

**Methods:**

A self-administered questionnaire survey was conducted with 337 SN from Japanese middle and high schools. The survey assessed SNs’ MHL and suicide literacy, including knowledge, attitudes, intended approaches, and confidence in addressing student mental health and suicide risks.

**Results:**

One-third of SN incorrectly believed that they could manage psychotic symptoms by careful listening alone. Many hesitated to ask students about suicide plans, even when risk was evident. Over half lacked confidence in providing mental health education.

**Conclusion:**

SNs’ MHL and suicide literacy are currently insufficient in Japan. Developing evidence-based training to improve these competencies is essential to strengthen school health services and promote better adolescent mental health and lower suicide risk.

## Introduction

The peak onset of many mental illnesses occurs during adolescence [1]. Mental illnesses among adolescents are associated with school refusal, academic decline, and social relationship problems, which can lead to impairments later in life [2,3]. Furthermore, mental illnesses are a major factor in suicide [4,5]. In the teenage years, suicide is a leading cause of death, being the number one cause of death among teenagers in Japan. Taking appropriate action before or during the onset of mental disorders is crucial for preventing symptom exacerbation and saving lives [1]. However, adolescents find it difficult to recognize their own mental health problems [6], and as the severity of mental health problems increases, there is a tendency to avoid seeking help from others [7,8]. In severe cases such as having suicidal ideation, there is a tendency not to seek help [5]. Merely waiting for adolescents to seek help themselves is insufficient to prevent the exacerbation of mental distress or suicide during adolescence; adults in their surroundings must actively notice, enquire and provide support.

Schools are commonly places where adolescent students can initially receive mental health support [9,10]. School nurses (SN) are often the most accessible health professionals in schools and can play a crucial role in recognizing students’ mental health issues, providing necessary support, encouraging help-seeking, and facilitating referrals to appropriate professionals [2,11–13]. However, the role of SN in school mental health is not always clearly defined, and the scope and quality of school health services differ considerably across countries, which may result in insufficient support for adolescents’ mental health needs [14,15]. In addition, insufficient training and support for healthcare professionals working with young people may be associated with adverse effects not only on the quality of care but also on their own mental health [16]. Recent research among hospital-based nurses suggests that mental health literacy remains suboptimal and may be associated with both nurses’ own mental health and their capacity to provide effective mental health support [17].

Given these challenges, clarifying and strengthening SNs’ literacy regarding adolescent mental health and suicide risk is essential. To address this need, a brief description of mental health literacy (MHL) and suicide literacy, as they relate to SN, is warranted. MHL refers to knowledge, attitudes, and skills regarding mental health [18]. Suicide literacy, a concept derived from MHL [19,20], refers to knowledge of risk factors for suicide and attitudes and responses to individuals with suicidal ideation, self-harm, or suicidal behaviour. In Japan, neither mental health nor suicide prevention training is mandatory during the licensure process for school nurses. Instead, such training opportunities are generally provided as voluntary continuing professional development after licensure.

To our knowledge, four studies have examined the MHL and suicide literacy of SN [21–24]. Among these, two studies examined SNs’ knowledge of and attitudes to depressive symptoms in students [23,24]. However, these studies did not focus on assessing the level of MHL among SN. Another study explored SNs’ perceptions of training needs related to posttraumatic stress disorder, depression with suicidal thoughts, and psychosis [21]. However, the questionnaire only asked about symptoms in adults (aged 24–37 years) and did not investigate mental disorders or suicide risk factors common among adolescents. One study examined knowledge and attitudes regarding suicide risk [22], but among 105 participants, only 26 were SN, and the results specifically for SN were not reported. Previous studies have not thoroughly examined the level of MHL and suicide literacy among SN regarding mental illnesses and suicide risk among adolescent students.

Therefore, this study aimed to investigate the MHL and suicide literacy of SN. The survey assessed SNs’ knowledge, recognition, attitudes to depression, schizophrenia, and social anxiety disorder (SAD), as representative mental illnesses during adolescence, and also their confidence in responding to students’ mental distress. Additionally, it evaluated SNs’ appropriate attitudes and understanding levels of factors contributing to suicide risk and students exhibiting suicide-related behaviours.

## Methods

### Study design and procedure

**Fig 1:**
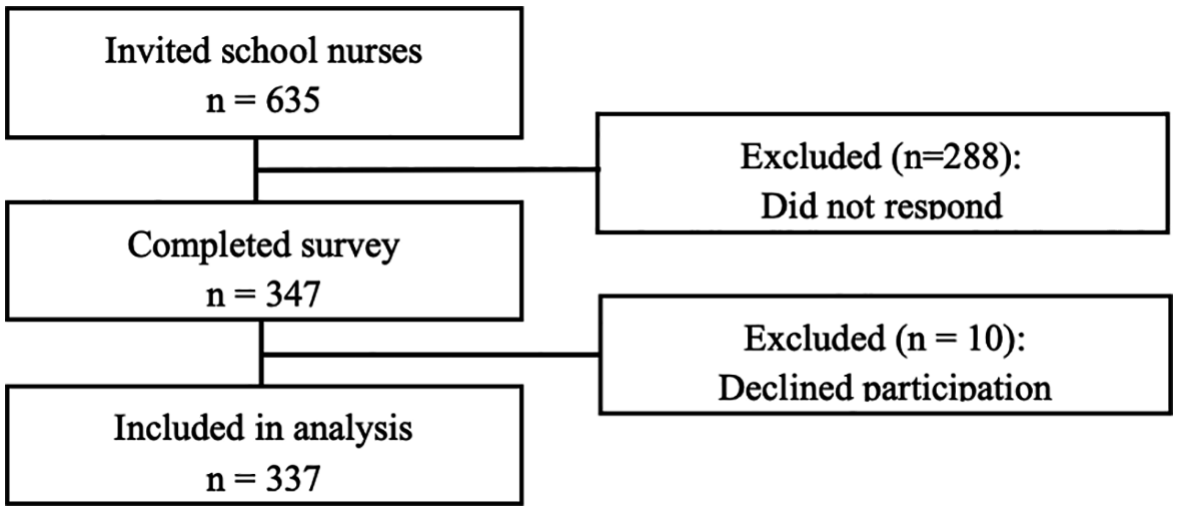
Participant flow diagram.

This study employed a cross-sectional design. Through a local education board in Japan, a notification regarding this study was sent to all public junior and senior high schools in Saitama prefecture, Japan, reaching a total of 635 SN. The prefecture was selected because it includes a large population with diverse characteristics, encompassing both urban and rural areas. Recruitment was conducted from 27/11/2020, and the survey was administered between January 2021 and March 2021. Of the SN contacted, 337 submitted consent forms and completed the survey, resulting in a participation rate of 53% (Fig 1). Reasons for non-participation were not collected. At the beginning of the questionnaire, the purpose and details of the study and the intended use of the data were explained in writing. SN were asked to indicate their willingness to participate by selecting “yes” or “no.” Those who answered “yes” proceeded to complete the survey, and this response was regarded as providing written informed consent. The study targeted professional SN and did not involve minors. The study protocol was approved by the Ethics Committee of the Graduate School of Education, The University of Tokyo (approval no. 18–48).

A total of 635 school nurses were invited to participate in the study.

Of these, 347 completed the survey, 10 declined participations, and 337 were included in the final analysis.

### Demographic characteristics of participants

Among the participants, 65% were aged 40 or above, and 54% had over 20 years of experience as SN. The majority held university degrees (74%), had experience in supporting students with mental disorders (87%), and had undergone mental health-related training in the past (79%). The sample size was based on the number of SN who voluntarily agreed to participate, and no formal sample size calculation was conducted.

### Assessment of mental health literacy (MHL) and suicide literacy

MHL and suicide literacy were evaluated using self-administered questionnaires. The questionnaires were developed and refined by a team of psychiatrists, psychologists, educators, and SN. The questionnaire consisted of seven parts:

#### Part 1: Demographic variables.

This section included questions regarding age, gender, school level (junior high or high school), years of experience as a school nurse, educational background, participation in mental health-related training, and experience of supporting individuals with mental disorders (Table 1).

**Table 1:** Demographic characteristics of participants.

Characteristics	n (%)
Age (years) 20-29 30-39 40-49 50-59 ≧60	56 (16.6) 62 (18.4) 80 (23.7) 118 (35.0) 21 (6.2)
Years of school nursing experience 10 or less 11 - 20 21 - 29 30 or more	73 (21.7) 81 (24.0) 80 (23.7) 101 (29.9)
Academic degree Bachelor’s Master’s (or higher) Not answered	250 (74.2) 44 (13.1) 43 (12.7)
Sex Female Male	335 (99.4) 2 (0.6)
School type Junior high school Senior high school	195 (57.9) 142 (42.1)
Previous participation in mental health seminars One or more times None	265 (78.6) 72 (21.4)
Experience of supporting a student suffering from mental illness Yes No	267 (87.0) 40 (13.0)

#### Part 2: Knowledge about mental health.

Basic knowledge about symptoms and treatments of mental illnesses commonly observed during adolescence, such as depression, schizophrenia, and SAD, was assessed using ten questions (see Table 2). The questions were based on Japan’s demographic statistics and were developed by the authors (a team including psychiatrists, psychologists, schoolteachers and SN). Topics were chosen to determine the extent to which SN understood the importance of attention to and care and prevention of mental illness in adolescents. Each item had three response options: “True,” “False,” or “Don’t know.” For the analysis, only correct responses (“True” or “False,” depending on the item) were counted as correct, while “Don’t know” was treated as incorrect. The internal consistency of the questions among participants in this study (Cronbach’s alpha coefficient) was 0.6.

**Table 2:** Knowledge about mental health in school nurses.

Item	Correct answer	Proportion corrects (%)
A majority of mental disorders begin to increase in prevalence during adolescence.	T	78.6
In Japan, 1 in 5 people will suffer from some kind of mental disorder in their lifetime.	T	50.1
With mental disorders, it is undesirable to return to school before treatment is fully completed.	F	67.7
Schizophrenia develops in 1 in 100 people.	T	52.8
More than 10% of people will suffer from depression in their lifetime.	T	59.0
In some cases of mental disorders, they may only cause physical symptoms such as headaches, stomachache or nausea.	T	83.7
By engaging in careful listening, it can be possible to cure hallucinations and persecutory delusions.	F	71.2
The duration of the treatment for depression and anxiety is usually more than a year.	T	61.4
Due to mental disorders, individuals may be unable to participate in daily conversations.	T	92.6
To reduce the risk of depression, 7 hours of sleep is best for high school students.	F	16.3

Note: n = 337; T = true; F = false; Proportions indicate the percentage of correct responses for each item.

#### Part 3: Perception of mental health symptoms in vignette cases.

Participants were presented with vignettes depicting symptoms of depression, schizophrenia, and SAD commonly seen in adolescents. The vignettes were developed by the authors (the same team as in part 2). The development of the case vignettes was informed by established diagnostic criteria and existing validated vignette formats. Specifically, the depressive vignette was developed with reference to the criterion A for major depressive disorder in DSM-5 [25] and the vignette format used in previous research [26]. Additionally, all vignettes were reviewed by a multidisciplinary expert panel consisting of psychiatrists, psychologists, schoolteachers, and experienced SN to ensure content validity and appropriateness for the Japanese school context. SN were asked to identify which symptoms corresponded with each disorder. The vignettes described adolescents exhibiting symptoms of the respective disorders (Table 3).

**Table 3:** School nurses’ recognition of mental health symptoms in vignette cases.

Correctly recognized the students’ symptoms as:	Proportion of correct responses (%)
Depression ^a)^	82.5
Schizophrenia ^a)^	82.5
SAD	83.4

Note: SAD = social anxiety disorder; ^a)^ Students A to C in the vignette cases (described in Methods, part 3).

The descriptions of the vignettes were as follows.

Depression: Student A complains of “headaches”, “stomachache” and “fatigue” and goes to the SNs’ office. A mentions, “I can’t sleep well, have no appetite, am unable to enjoy my favourite TV shows, and I can’t concentrate on my studies.” Recently, A’s tardiness has been increasing.

Schizophrenia: Student B appears to be unable to concentrate during classes. B covers her/his ears during break times. When you (SN) ask B about her/his condition, B mentions, “Everyone in the class is talking about me. Someone is always watching me. Strangers passing by say hurtful things to me. I’m extremely bothered by the situations surrounding me.”

SAD: Student C is extremely introverted. C can talk with her/his family, but cannot communicate well with anyone else, and struggles with communication with classmates. C can hardly make friends even though she/he wants to. During presentations in her/his class, C becomes nervous, blushes, and her/his voice may tremble. C is always worried that classmates or others may perceive her/his behaviour to be strange.

#### Part 4: attitude to students with mental health problems.

Using the same vignettes as in Part 3, participants were asked about their agreement or disagreement with statements reflecting attitudes to students with mental health problems (Table 4).

**Table 4:** School nurses’ attitude to mental health problems in vignette cases.

	Disorder	Agree (%)
*The student’s problem is not a matter for medical treatment.*	Depression	2.4
	Schizophrenia	2.1
	SAD	12.7
*If the student desires, she/he can easily overcome this state.*	Depression	16.9
	Schizophrenia	3.9
	SAD	22.2
*The student’s problem is due to personal weakness.*	Depression	2.7
	Schizophrenia	0.9
	SAD	3.6
*To avoid becoming like the student, other students should not associate with her/him.*	Depression	0
	Schizophrenia	1.2
	SAD	0
*Students like in the vignette case are unpredictable in their actions.*	Depression	41.9
	Schizophrenia	65.9
	SAD	10.7

Note: SAD = social anxiety disorder. Values indicate the percentage of school nurses who agreed with each statement.

#### Part 5: confidence in supporting students with mental health problems.

Participants were asked to rate their confidence level in providing support and assistance to the students depicted in the vignettes (Table 5).

**Table 5:** School nurses’ confidence regarding students with mental health problems.

Case / topic	Not at all	Not much	A little	Enough
Supporting student A (depression)	0.6	30.3	63.8	5.3
Supporting student B (schizophrenia)	4.2	49.0	43.6	3.3
Supporting student C (social anxiety disorder)	0.9	31.2	63.8	4.2
Instructing students about mental health problems	6.5	55.8	36.5	1.2

Note: Percentages indicate responses to each question. For vignette items (students A to C), participants were asked: “How confident would you be in providing the necessary support?”

#### Part 6: knowledge about suicide risks.

Basic knowledge about the epidemiology, risk factors, and care/treatment of suicidal ideation/behaviour among adolescents was assessed using ten questions (Table 6). This part was also drafted in the same manner as Part 2, and the internal consistency among participants in this study (Cronbach’s alpha coefficient) was 0.55.

**Table 6:** School nurses’ knowledge about preventing suicide.

Item	Correct answer	Proportion correct (%)
Asking about suicidal ideation should be avoided, because it can lead to suicide attempts.	F	83.7
In Japan, suicide is the first leading cause of death in late teens.	T	82.2
Paying attention to students who repeatedly self-harm is the most important in suicide prevention.	F	53.7
Asking about suicidal thoughts should be left to experts, and teachers/school nurses should not ask students about such thoughts.	F	81.6
Risk of suicide does not differ depending on whether a person has someone to discuss their worries with or not.	F	86.9
Attention to suicide prevention in someone should be at the same level whether the person has a history of suicide attempts or not.	F	23.7
If you were told a suicide plan and were begged, “Don’t tell anyone about it”, you should not tell anyone about it.	F	94.9
The focus in suicide prevention should be primarily on students being treated by psychiatrists.	F	67.9
When teachers/school nurses examine the risk of suicide, they should not ask whether the student has a specific plan to commit a suicide or not.	F	53.7
If suicide risks are very high, the student should be hospitalized, even if she/he does not agree.	T	54.8

Note: n = 337; T = true; F = false; Proportions indicate the percentage of correct responses for each item.

#### Part 7: Intended Approach to Suicidal Ideation (SI) and suicide plans.

The content of the vignette is about a teenage student who has suicidal thoughts. The vignette modified from a previous study [27] posed the question, “If you were in a position where you interacted with the vignette student on a regular basis, would you immediately take subsequent action?”, SN were asked to respond on a four-point Likert scale of “strongly agree”, “agree”, “disagree”, “strongly disagree”. The description of the vignette was as follows.

*Suicidal Ideation / Behaviour*: “Student D feels that she/he will never be happy again and believes that her/his family would be better off without her/him. She/He has been so desperate, and she/he has been thinking of ways to end her/his life.”

Having read this vignette, SN were asked to what extent they agreed with the 5 items regarding “intention to ask students about their suicidal thoughts and plans”, and to respond on a four-point Likert scale of “strongly agree” to “strongly disagree” (see Table 7).

**Table 7:** Intention to ask about suicidal thoughts and plans.

Item	Strongly agree	Agree	Disagree	Strongly disagree
Ask whether the student does not want to live	38.6	38.3	14.2	8.9
Ask whether the student wants to die	36.5	36.5	13.9	13.1
Ask whether the student has ever thought about how to commit suicide	32.3	34.4	21.7	11.6
Ask whether the student has prepared for suicide	31.5	35.9	21.4	11.3
Ask whether the student has actually done something or been somewhere to die	34.1	38.3	17.2	10.4

Note: n = 337. Values are percentages of responses to each item.

### Data analysis

Descriptive statistics including frequencies, percentages, means, and standard deviations were calculated. Multiple regression analyses were conducted to examine the relationship between mental health/suicide literacy and experience, with mental health knowledge (described in Part 2) and suicide risk knowledge (Part 6) as dependent variables and years of experience as an independent variable. Logistic regression analyses were performed to explore the relationship between confidence/intended approach and experience/literacy, with confidence in instructing students about dealing with mental health problems (Part 5) or intention to ask students about suicide (Part 7) as the dependent variables and years of experience/mental health knowledge/suicide risk knowledge as independent variables. Confidence in instructing students about dealing with mental health problems was dichotomized into “having confidence” (including answer options “A little” and “Enough”) and “lacking confidence” (including answer options “Not at all” and “Not much”). Intention to ask students about suicide was dichotomized into “intending to ask” (including answer options “Strongly agree” and “Agree”), and “Not intending to ask” (including “Strongly disagree” and “disagree”). The analysis was performed using R version 4.1.3.

Data were collected anonymously and on a voluntary basis, which may have helped mitigate potential biases such as social desirability bias and self-selection bias inherent in self-administered surveys. Cases with missing data were excluded from the analysis. Therefore, all variables included in the analyses had complete data.

## Results

### Knowledge about mental health (Table 2)

Approximately 1 in 5 participants did not know that “A majority of mental illnesses begin to increase in prevalence during adolescence” (79% correct). The correct response rate to the statement, “In Japan, 1 in 5 people will suffer from some kind of mental disorder in their lifetime” was 50%. Approximately 1 in 3 participants incorrectly believed that “With mental illnesses, it is undesirable to return to school before treatment is fully completed” (68% correct) and incorrectly thought that “By engaging in careful listening, it can be possible to cure hallucinations and persecutory delusions” (71% correct). Approximately 40% were unaware that “The duration of the treatment for depression and anxiety is usually more than a year” (61% correct). The item with the lowest correct response rate was “To reduce the risk of depression, 7 hours of sleep is best for high school students” (16% correct).

### Perception of mental health symptoms in vignette cases (Table 3)

The identification rates for symptoms corresponding to depression, schizophrenia, and SAD in vignette cases depicting adolescent students suffering from mental illness were approximately 80% for each disorder. In other words, 1 in 5 participants were unable to distinguish the symptoms of mental illness.

### Attitude to students with mental health problems (Table 4)

With vignettes depicting symptoms of depression and schizophrenia, only a small percentage (2%) incorrectly agreed that these disorders are “not a matter for medical treatment”. However, with vignettes about SAD, 13% believed this not to be a matter for medical treatment. Regarding the item, “If the student desires, she/he can easily overcome this state”, 22% agreed for SAD, 17% for depression and 4% for schizophrenia. Only a small percentage of participants agreed with the statement, “The student’s problem is due to personal weakness” (3% for depression, 1% for schizophrenia, 4% for SAD). Regarding the item, “To avoid becoming like the student, other students should not associate with her/him”, 0% agreed for depression and social anxiety disorder, and 1% for schizophrenia.

### Confidence in supporting students with mental health problems (Table 5)

SNs’ confidence in providing support to students exhibiting symptoms of mental illnesses (depression, schizophrenia, SAD) was as follows: 31% lacked confidence for depression, 53% for schizophrenia, and 32% for SAD. Regarding confidence in instructing students about dealing with mental health problems, 62% lacked confidence.

### Knowledge about suicide risks (Table 6)

Approximately half of the participants (54%) incorrectly believed that “when assessing suicide risk, it is not appropriate to enquire whether the student has a specific plan for committing suicide.” About 1 in 5 SN incorrectly believed that “Asking about suicidal thoughts should be left to experts, so teachers/school nurses should not ask students about such thoughts” (82% correct).

### Intention to ask students about suicidal thoughts and plans (Table 7)

Between 1 in 5 and 1 in 3 participants expressed disagreement about asking about suicidal thoughts or plans regarding the student in the vignette case. Approximately 27% disagreed with asking whether the student wanted to die, 33% disagreed with asking whether the student had considered committing suicide, and about 28% disagreed with asking whether the student had taken steps or been somewhere with the intent to die.

### Mental health / suicide literacy and experience (Table 8)

There was no significant trend of increasing knowledge level with more years of experience. There was no statistically significant relationship between MHL and years of experience. For knowledge scores related to suicide risk, there was a trend of lower scores among SN with more years of experience, which was statistically significant (p < 0.05).

**Table 8:** Regression results for school nurses’ mental health and suicide literacy.

Dependent variable	Independent variable	B	SE	z	p	95% CI	OR
Confidence in instructing MHL	Experience	0.05	0.01	3.96	* p < .001.	[0.02, 0.07]	1.05
	MHL	0.28	0.06	4.48	* p < .001.	[0.16, 0.41]	1.32
Intention to ask about suicidal thoughts	Experience	−0.03	0.01	−2.61	* p < .001.	[-0.06, -0.01]	0.97
	Suicide literacy	0.41	0.07	5.49	* p < .001.	[0.27, 0.56]	1.51

Note: B = unstandardized regression coefficient; t = t-value; p = probability value; SE = standard error; CI = confidence interval.

a)Individual scores for Mental Health Literacy; Knowledge about Mental Health (Table 2)

b)Individual scores for Knowledge about Suicide Prevention in Adolescents (Table 6)

c)Years of school nursing experience

### Confidence / intended approach and experience, literacy (Table 9)

Regarding the item, “Confidence in instructing students about dealing with mental health problems”, responding as “confident” (choosing either “a little” or “enough”) was significantly associated with both years of experience (odds ratio (OR) 1.05) and knowledge (OR 1.32), both of which were statistically significant (p < 0.001). Additionally, the intention to enquire about suicidal ideation among students was significantly associated with both years of experience (OR 0.97) and knowledge (OR 1.51), with more years of experience correlating with lower intention (p < 0.001).

**Table 9:** Regression results for school nurses’ confidence and intended approach in relation to experience and literacy.

Dependent variable	Independent variable	B	SE	t	p	95% CI
MHL ^a)^	Experience ^c)^	−0.0007	0.01	−0.07	p = .95	[-0.02, 0.02]
Suicide literacy ^b)^	Experience ^c)^	−0.02	0.01	−2.52	* p < .05.	[-0.04, -0.005]

Note: B = unstandardized regression coefficient; z = z-value from logistic regression analysis; p = probability value; SE = standard error; CI = confidence interval; OR = odds ratio.

## Discussion

This study revealed that SN have insufficient MHL and suicide literacy, and many SN lack confidence in supporting students with mental illness. SN with high mental health knowledge tend to have more confidence in instructing students how to deal with mental disorders, while SN with high suicide risk knowledge are more likely to have the intention to enquire about suicidal thoughts in suicidal students. These findings suggest the necessity of training SN to equip them with accurate knowledge and confidence to appropriately address the mental health and suicide risks of adolescents.

Approximately 1 in 3 SN mistakenly believed that it is undesirable for students suffering from mental illness to return to school before completing treatment, or that by engaging in careful listening, it can be possible to cure hallucinations and persecutory delusions. Additionally, about 1 in 5 SN did not know that many mental illnesses begin to increase during adolescence. This lack of knowledge can lead to the oversight of mental illnesses and create barriers to students receiving appropriate care. Furthermore, many SN believed that seven hours of sleep is sufficient for high school students, indicating that lifestyle guidance to prevent mental illness may not be appropriately provided.

It was found that many SN have insufficient knowledge and awareness regarding adolescent suicide risks. About half of the SN mistakenly believed that it is better not to ask about suicide plans, thinking it is the job of specialists, and approximately 1 in 3 SN disagreed with asking about suicidal ideation in students with suicidal thoughts or behaviours. SN serve as key gatekeepers who can identify suicide risk and facilitate referrals and follow-up support in collaboration with professionals [28]. However, youth suicide is often difficult to detect due to a lack of overt warning signs [29], and in Japan, more than 70% of students who died by suicide had shown no explicit suicidal intent prior to death [30]. Therefore, early and proactive risk assessment in schools is essential, and strengthening SNs’ suicide literacy is crucial to prevent student suicide.

There is room for improvement in SNs’ attitudes to mental illness. 1 in 5 SN believe that individuals with SAD can easily overcome their condition if they desire to do so, while 1 in 6 holds the same belief regarding depression. Additionally, more than 1 in 10 SN do not consider SAD to be a condition that requires medical treatment. SN play a crucial role in addressing the mental health needs of students, not only within school settings but also by collaborating with community partners and general practitioners [31,32]. However, if the attitude that mental illness is not a medical issue persists, SN may be unable to fulfil their role effectively, as they may fail to refer students for medical intervention when this would be advantageous for student outcomes. Insufficient MHL among frontline professionals is not unique to Japan. For example, about half of community-based primary healthcare workers in Nepal demonstrated limited MHL, and higher educational attainment and mental health training were positively associated with better literacy [33]. In the UAE, less than half of school nurses correctly identified mental health conditions such as PTSD, depression with suicidal ideation, and psychotic disorders in vignette-based assessments—although these vignettes depicted adults—and evidence-based treatment selection was also insufficient [21]. While healthcare and educational systems differ across countries, a common challenge is that professionals positioned as accessible gatekeepers for adolescents do not always possess adequate MHL. These findings underscore the international need for practical and context-appropriate training programs tailored to the specific roles and environments of school-based personnel. Cross-national comparisons provide contextual grounding for the present findings and support the broader relevance of strengthening MHL among SN internationally.

Furthermore, in this study, higher suicide literacy was significantly associated with a stronger intention to enquire about suicidal ideation (p < 0.001, OR 1.51), and higher mental health knowledge was associated with greater confidence in providing support (p < 0.001, OR 1.32). In contrast, more years of experience were not necessarily linked to higher levels of MHL. A previous study among basic healthcare providers and community health volunteers also reported that longer experience does not guarantee up-to-date MHL [33], suggesting that this may be a shared challenge for SN as well. Therefore, rather than relying solely on experience, it is important to strengthen up-to-date knowledge and confidence (competence) through structured education and ongoing training.

This study highlights the need to develop training programmes that address the identified knowledge gaps: how SN should interact with students with mental disorders, the necessary medical and epidemiological knowledge, correct information about preventive lifestyles for mental disorders, and education to deepen the understanding of the importance of asking about specific suicide plans and understanding risk factors. Continuous verification and improvement of these programmes are desired to ensure their effectiveness.

### Limitations

This study has several limitations. First, the participants were limited to SN in a single prefecture in Japan, which may limit the generalizability of the findings. Although this prefecture includes both urban and rural areas and has a relatively large population, caution is required when generalizing the findings to other regions and countries. Second, the response rate was 53%, which is not low compared with previous studies [21–24], but the potential for non-response bias cannot be excluded.

Third, the questionnaire used in this study was developed by a multidisciplinary research team including psychiatrists, psychologists, schoolteachers, and SN, based on the practical context of Japanese school settings and by referring to DSM-5 criteria [25]. Therefore, it is not an internationally standardized measure, and direct comparison with studies conducted in other countries may be limited. However, previous studies have also not used a single standardized tool to comprehensively assess both MHL and suicide literacy. For example, knowledge, attitudes, and skills related to depression among SN have been independently assessed using study-specific items [21,23,24], while suicide literacy has often been measured based on experience, confidence, and participation in suicide-specific training [22]. In addition, studies among basic healthcare providers have developed new MHL measures incorporating suicide risk recognition [33], highlighting the lack of an internationally standardized and integrated scale for evaluating both constructs. Taken together, the development of an assessment framework that enables international comparison remains an important future task. The present study may contribute foundational knowledge toward developing such internationally comparable measures and identifying essential literacy components required for SN across different countries.

### Implications

The findings of this study hold important implications for school-based suicide prevention and mental health support. SN are expected to recognize early signs of emotional difficulties and respond appropriately within the school context. Prior studies have indicated that insufficient awareness of symptoms and available services can delay adolescents’ help-seeking and access to care [34], and SN often serve as the most accessible health professionals for students [11]. Therefore, strengthening SNs’ mental health literacy and suicide literacy can facilitate the early detection of mental health problems and timely connection to appropriate support. Furthermore, equipping SN with adequate skills and confidence may help strengthen the school’s capacity to provide timely support, contributing to a more comprehensive and sustainable school mental health system. Although preliminary, the assessment framework developed in this study may offer useful insight into the essential literacy components required for SN and provide a basis for developing evaluation tools that could eventually be applied in international comparisons. These findings may also inform the gradual integration of MHL components into existing school health activities, such as staff training, health education, and multidisciplinary collaboration.

## Conclusion

This study comprehensively evaluated SNs’ mental health literacy and suicide literacy based on the practical context of Japanese school settings. The findings revealed insufficient knowledge of symptoms, risk factors, and preventive lifestyles related to mental illness, limited confidence in providing support, and a low intention to directly enquire about suicidal ideation. These gaps indicate areas that need to be addressed for SN to effectively contribute to the promotion of students’ mental health and the prevention of suicide. In addition, the findings suggested that longer years of experience do not necessarily lead to higher literacy levels, highlighting the limitations of relying solely on routine work to maintain and improve professional competence. Given that SN are the most accessible health professionals for students within schools, their role in early identification of problems and appropriate referral is essential for school-based prevention of mental illness and suicide. Therefore, boards of education and training institutions, including universities responsible for preparing SN, should develop systematic and continuous training programmes aimed at improving mental health literacy and suicide literacy among SN based on the gaps identified in this study. Such programmes may support daily school-based practices, including early identification of mental health problems, direct inquiry about suicidal ideation, information sharing among school staff, and appropriate referral to support services. Furthermore, the assessment framework used in this study provides preliminary insights into the essential literacy components required for SN. Future research should focus on developing and evaluating training programmes that address these insufficient areas. In addition, further sharing of knowledge and practices across domestic and international contexts may contribute to advancing the role of SN in school-based mental health support.
